# The day‐to‐day stability of the ruminal and fecal microbiota in lactating dairy cows

**DOI:** 10.1002/mbo3.990

**Published:** 2020-03-16

**Authors:** Shuai Huang, Shoukun Ji, Hui Yan, Yangyi Hao, Jun Zhang, Yajing Wang, Zhijun Cao, Shengli Li

**Affiliations:** ^1^ State Key Laboratory of Animal Nutrition Beijing Engineering Technology Research Center of Raw Milk Quality and Safety Control College of Animal Science and Technology China Agricultural University Beijing China; ^2^ College of Animal Science and Technology Hebei Agricultural University Baoding China

**Keywords:** bacterial community, dairy cow, feces, rumen fluid

## Abstract

In this study, we examined differences between the microbiota of the ruminal fluid (DR) and feces (DF) from five lactating dairy cows over three consecutive days using 16S rRNA gene sequence‐based analysis. Results showed significant differences between the microbial communities of the DR and DF. In particular, the relative abundance of the phyla *Firmicutes* and *Actinobacteria* was significantly lower (*q* < 0.001) in DR compared with DF, while the relative abundance of *Bacteroidetes* was significantly higher in DF than that of DR (*q* < 0.001). A significantly higher relative abundance of the genera *Bifidobacterium*, *5‐7N15*, *Clostridium*, *Epulopiscium*, *SMB53*, *Turicibacter*, *Dorea*, *Roseburia*, and *Akkermansia* was observed in the DF, while a higher relative abundance of the genera *Prevotella*, *Butyrivibrio*, *CF231*, *RFN20*, and *Succiniclasticum* was observed in the DR. A further analysis using the functional prediction program PICRUSt showed that sequences belonging to the *5‐7N15*, *Akkermansia*, *Bifidobacterium*, *Clostridium*, *Dorea*, *Epulopiscium*, *Roseburia*, and *Turicibacter* were significantly and positively correlated with glycan biosynthesis and metabolism, while *CF231*, *Prevotella*, *RFN20,* and *Succiniclasticum* were significantly and positively correlated with amino acid, lipid, carbohydrate, other amino acid, cofactors, and vitamins metabolism. No significant differences were observed across the three consecutive days in either the DR or DF ecosystems, with no significant differences in the diversity or abundance at the phylum and genus levels suggested that there is a limited day‐to‐day variability in the gut microbiota.

## Graphical abstract

This study compared differences between the rumen fluid and fecal microbiota of dairy cows and further sought to determine whether differences exist between individual samples collected over three consecutive days. A significant difference was found between the rumen and fecal microbiota suggesting that dairy cows have different microbial communities between ruminal fluid and feces. Notably, no differences were observed over the three days sampling, indicating that the gut microbial community of dairy cows may be likely stable on a day‐to‐day basis.

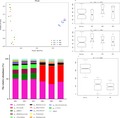

## 1 INTRODUCTION

Gastrointestinal bacterial communities perform vital functions in the mammalian gut. In ruminants, ruminal bacteria ferment plant fibers to produce volatile fatty acids and bacterial proteins, which are subsequently absorbed and metabolized to produce meat or milk (Mackie, 2002). Fermentation of feed material also occurs in the hindgut (Vanhatalo & Ketoja, 1995), producing various metabolites and providing important vitamins for the host (Godoy‐Vitorino et al., 2012). Although a sizable body of literature is available on the role of rumen fermentation in ruminants, relatively less is known about the hindgut fermentation process in dairy cows.

Because feed presented to the rumen and hindgut may differ, the microbiota in the rumen and feces are likely also distinct. Mao, Zhang, Liu, and Zhu (2015) analyzed the bacterial communities along the gastrointestinal tract in six lactating dairy cows and found significant differences in microbial community composition, including species diversity and abundance. In a different study, Liu, Zhang, Zhang, Zhu, and Mao (2016) reported decreased species diversity in fecal microbiota compared with rumen microbiota. Similarly, de Oliveira et al. (2013) found overt differences in microbial communities between the rumen and feces of Brazilian Nelore steers. The microbial composition of the ruminal fluid and feces can be further affected by other factors such as diet (McCann, Wickersham, & Loor, 2014). Thus, dissimilarities among diets and sampling sites may hinder analyses of these bacterial communities.

In addition, while some studies have examined representative microbiota samples from ruminal fluid or feces collected at a single time point, many other studies have used samples collected over three consecutive days (Shabat et al., 2016; Zhou et al., 2018). Based on 16S rRNA sequencing techniques, Skarlupka, Kamenetsky, Jewell, and Suen (2019) found a limited day‐to‐day variability in the rumen microbiota from three consecutive days. However, the work carried out by Skarlupka et al. (2019) was mainly focused on the rumen microbiota and did not explore the difference in the fecal microbiota on a day‐to‐day basis. Thus, we characterized the microbial communities of ruminal fluid and feces from five lactating dairy cows over three consecutive days to examine differences in bacterial communities among sampling days.

## 2 MATERIALS AND METHODS

### 2.1 Animals and sample collection

Five five‐year‐old lactating Holstein dairy cows were housed in a free barn at a commercial dairy farm (Beijing, China) and were cared for according to the practices outlined in the Guide for the Care and Use of Agricultural Animals in Agricultural Research and Teaching (FASS, 2010). Cows were fed a total mixed ration ad libitum, formulated to meet or exceed the energy requirements of lactating dairy cows (NRC, 2001) (Table A1). The cows were adapted to the diet and barn for at least 40 days prior to sample collection. The cows had free access to fresh water. During this period, none of the cows experienced disease and did not receive any antibiotic treatment.

DR was collected via the mouth prior to morning feeding on three consecutive days using a stomach tube with a rumen vacuum sampler (A1141K, Ancitech, Winnipeg, CA). DR samples were filtered through a four‐layer cheesecloth. Fecal samples were obtained from the rectum prior to morning feeding on three consecutive days using sterile gloves. All samples were immediately frozen in liquid nitrogen and stored at −80°C until further analysis.

### 2.2 DNA extraction, PCR amplification, and Illumina MiSeq sequencing

Total DNA was extracted from the DR and DF samples using a Qiagen DNA Extraction Kit (Qiagen, Hilden, Germany). DNA samples were quantified using a NanoDrop ND‐1000 Spectrophotometer (NanoDrop Technologies, Wilmington, DE, USA) and then stored at −80°C until being used as a template for the PCR assays. Barcoded primers 343F (5ʹ‐GATCCTACGGGAGGCAGCA‐3ʹ) and 534R (5ʹ‐GCTTACCGCGGCTGCTGGC‐3ʹ) were used to amplify the V3‐V4 region of the 16S rRNA gene (Ji et al., 2017). PCR reactions for each sample were carried out on a Mastercycler Gradient (Eppendorf, Germany) using 25 μl reaction volumes containing 2 μL template DNA, 5 μM of each forward and reverse primers, 12.5 μL 2 × Taq PCR MasterMix (KAPA Biosystems, Wilmington, USA), 3 μL BSA(2ng/μl), and 5.5 μL ddH_2_O. PCR assays consist of an initial denaturing step at 95°C for 5 min followed by 32 cycles of 95°C for 45 s, 55°C for 50 s, and 72°C for 45 s with a final extension at 72°C for 10 min. Samples were run on a 2% agarose gel using electrophoresis, and those samples with a band appearing between 200 and 210 bp were extracted and purified using a QIAquick Gel Extraction Kit (QIAGEN, Germany).

Following qualification and quantification, purified amplicons were pooled at equal molarity and sequenced on an Illumina MiSeq platform using a MiSeq Reagent Kit V3 (600‐cycle, Illumina, San Diego, CA, USA) according to standard protocols (Caporaso et al., 2012).

### 2.3 Taxonomic classification and diversity analysis

Shorter reads (lower than 200 bp), low‐quality sequences (scores lower than 20), ambiguous bases, and chimeras were removed by USEARCH in the QIIME1 pipeline (version 1.5.0) (Caporaso et al., 2011). Clean, paired‐end sequence reads were merged using FLASH (version 1.2.7) (Magoc & Salzberg, 2011), and 16S rRNA sequences were classified using UCLUST (version 1.2.22) against the SILVA bacterial database (SILVA version 119, released in July, 2014) (Pruesse et al., 2007). Operational taxonomic units (OTUs) determined at 97% similarity was carried out using UCLUST (Edgar, 2010). All singletons and doubletons were removed using UCLUST to generate a representative OTU table. To ensure even sequencing depth across samples, the number of tags per sample was randomly subsampled to 15,900 for bacterial community analysis.

### 2.4 Statistical analysis

Alpha diversity analysis was performed using QIIME including Chao 1 index calculation, determination of the number of OTUs, phylogenetic diversity whole tree analysis, and Shannon's diversity index were calculated from the OTU table. Beta diversity indices were measured based on unweighted UniFrac distances and displayed using principal coordinate analysis (PCoA) (Lozupone & Knight, 2005). Analysis of similarities (ANOSIM) was performed to determine the overall difference in microbial communities by sampling sites using the Vegan package (Oksanen et al., 2015) in R (version 3.1.2). Functional classification was predicted using Phylogenetic Investigation of Communities by Reconstruction of Unobserved States (PICRUSt, https://huttenhower.sph.harvard.edu/galaxy) from 16S rRNA sequencing, and Kyoto Encyclopedia of Gene and Genomes (KEGG) functional composition profiles were generated (Langille et al., 2013). Spearman's rank correlation analysis was used to analyze the correlation between predicted functional profiles and the genus (relative abundance of more than 1%).

Specific bacterial taxa were tested using the Kruskal–Wallis analysis to assess differences in relative abundance on different sampling days within the same community, while the Wilcoxon rank‐sum test was used to determine differences in relative abundance at the phylum and genus levels between communities. Statistical analysis was performed in R (version 3.1.2), with statistical significance accepted at *p < *.05. All *P*‐values from the multiple comparison analyses were adjusted based on the false discovery rate (Benjamini & Hochberg, 1995), with statistical significance accepted at adjusted *q*‐values < 0.05.

## 3 RESULTS

### 3.1 Differences in bacterial community diversity and composition between sampling sites and over different days

**Figure A1:**
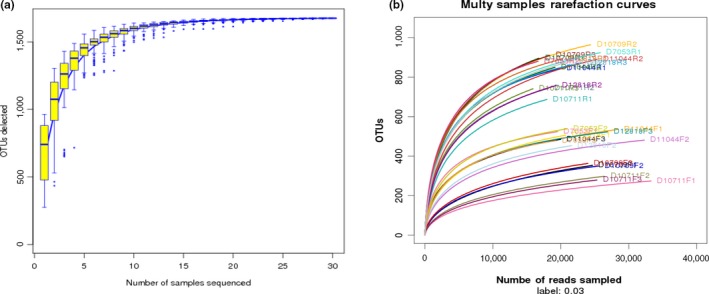
Rarefaction analysis for assessing OTU coverage. (a) Sample‐based rarefaction curve showing the increase in OTU numbers as a function of the number of individuals sampled. Each added sample adds OTUs to the plot, which has not yet been seen in previous samples. The curve becomes asymptotic as the OTU number saturates, and each sample adds an increasingly smaller number of new OTUs, indicating adequate coverage for the environment being tested. (b) Individual rarefaction curves for each rumen sample taken.

Our 16S rRNA gene sequencing produced an average of 22,332 ± 4,411 high‐quality sequences for each of the 30 samples in our study. Based on a sequence similarity cutoff of 97%, a total of 1,679 OTUs were detected across all samples. Rarefaction analysis was conducted to assess OTU coverage, producing a Good's coverage value > 0.97 (Figure A1) for each sample, implying that the sequence coverage was sufficient. Chao 1, number of OTUs, phylogenetic diversity whole tree analysis, and Shannon diversity index values for DR were significantly higher than those obtained for DF (*q < *0.001). Importantly, no differences in these values were observed for either community across different sampling days (*q > *0.05) (Table 1).

**Table 1:** Alpha diversity indices for the DR and DF bacterial communities

Items	Feces^a^			Rumen^b^			*SEM*	*q* value
	DF1	DF2	DF3	DR1	DR2	DR3		
Chao1	515.08^c^	488.26^c^	495.16^c^	951.24^d^	929.33^d^	960.30^d^	44.20	＜0.0001
Shannon	4.88^c^	4.51^c^	4.57^c^	7.13^d^	7.04^d^	7.16^d^	0.26	＜0.0001
Number of OTUs	381.20^c^	361.40^c^	372.80^c^	789.60^d^	770.60^d^	791.60^d^	41.31	＜0.0001
PD_whole_tree	34.62^c^	33.03^c^	33.58^c^	62.39^d^	60.23^d^	61.82^d^	2.74	＜0.0001

a Feces: DF1, feces samples from day 1; DF2, feces samples from day 2; DF3, feces samples from day 3.

b Rumen: DR1, rumen samples from day 1; DR2, rumen samples from day 2; DR3, rumen samples from day 3.

^cd^ Different letters within a row indicate a significant difference between values (*q < *0.05).

**Figure 1:**
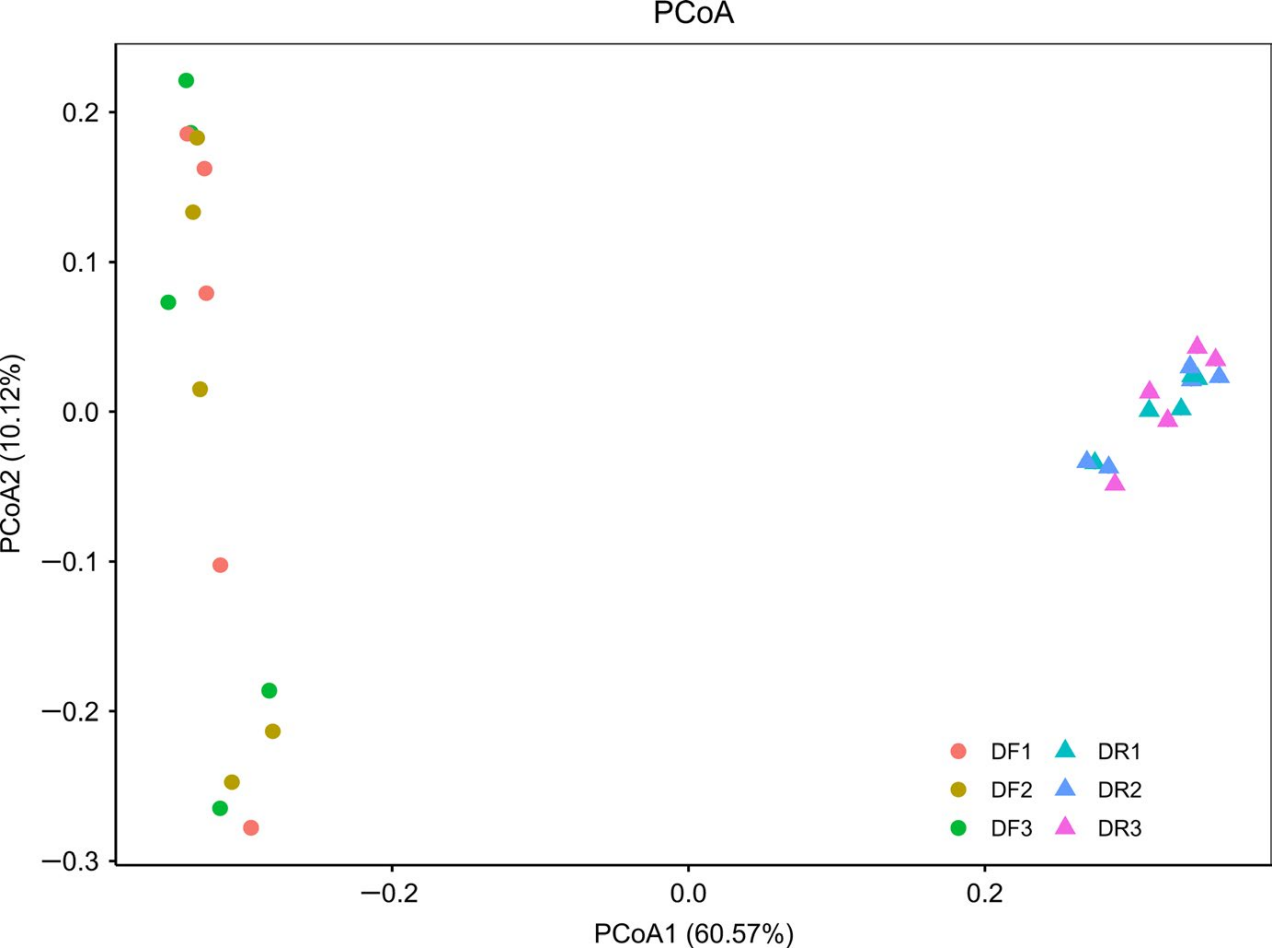
PCoA plot for comparing bacterial communities from the different gut sections and different days based on unweighted UniFrac distance matrices

**Figure 2:**
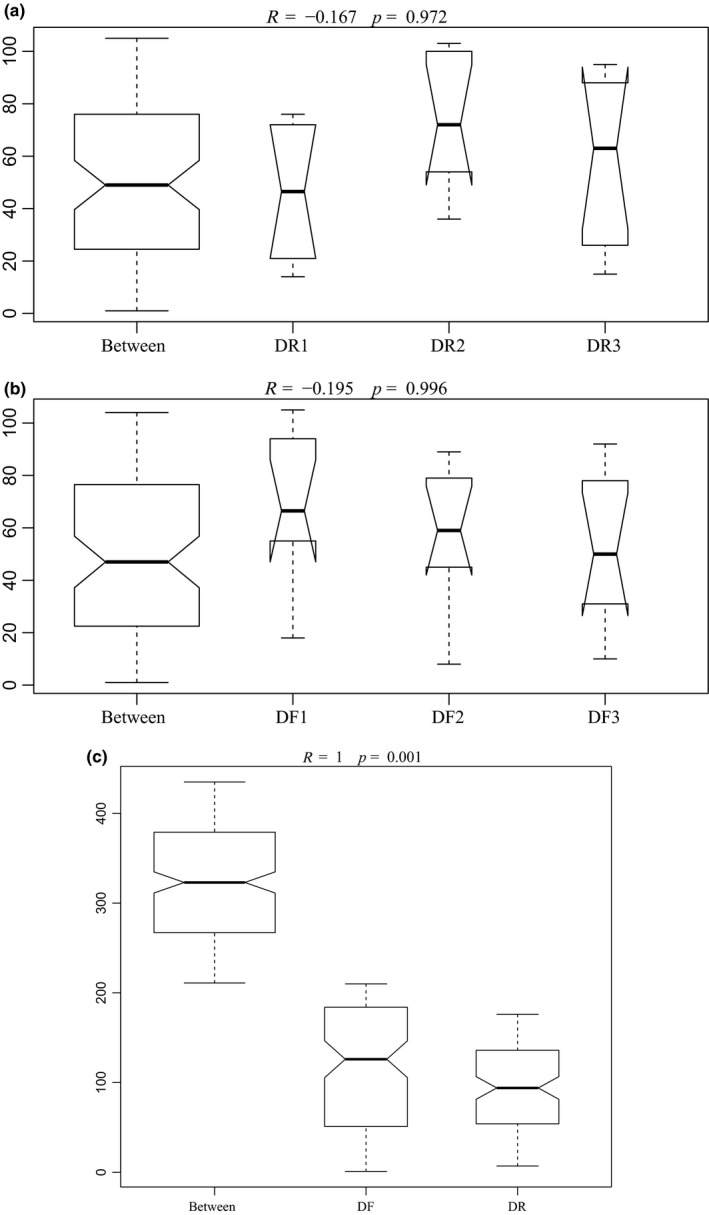
ANOSIM analysis of the different samples. ANOSIM results are presented as a box plot, where bacterial communities are grouped by sampling time in ruminal fluid (a) and feces (b) and by gut location (c). The analysis was conducted using a Bray–Curtis metric based on operational taxonomic units. DF indicates fecal microbiota samples; DR indicates ruminal fluid microbiota samples

We then used a PCoA to examine difference in microbial community structure. While there was an obvious separation of the DR and DF bacterial communities, samples from the same community collected over the three consecutive days clustered together (Figure 1). Our ANOSIM analysis produced *R*‐values of < 0 and *p*‐values > 0.05, implying that there were no significant differences between sampling days for either the DR (*R* = −0.167, *p = *.972) (Figure 2a) or DF (*R* = −0.195, *p = *.996) microbiota (Figure 2b). In comparison, statistically significant differences between the DR and DF (*q = *0.001, Figure 2c) were obtained by ANOSIM analysis.

### 3.2 Bacterial community composition

**Figure A2:**
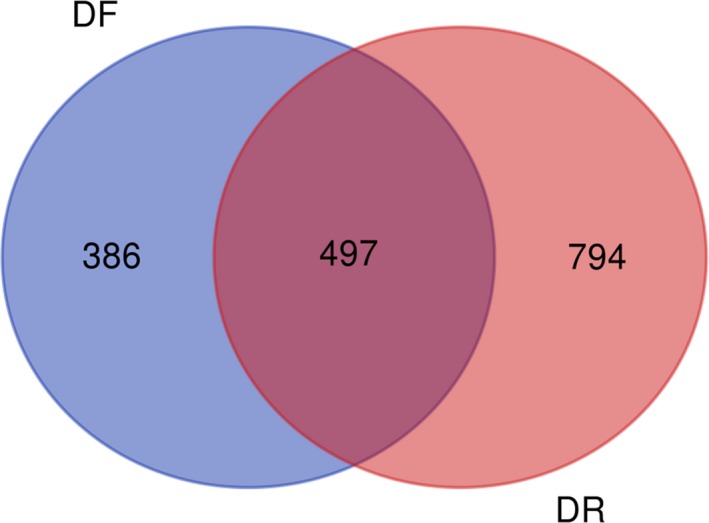
Venn plot for shared OTUs.

Only 497 of the 1,679 identified OTUs were shared between the DF and DR bacterial communities (Figure A2). Overall, 883 OTUs were specific to the DR samples and 386 OTUs were specific to the DF samples (Figure A2).

**Figure 3:**
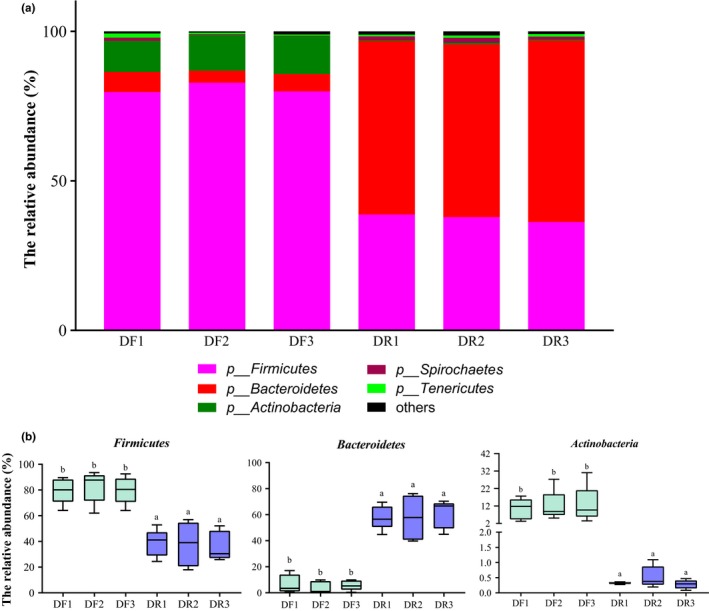
Distribution of predominant phyla (a) and the abundance of significantly different phyla (b). ^ab^Different letters within a row indicate a significant difference (q < 0.05)

A total of 17 phyla were identified across all samples. The bacterial communities from all samples were dominated by the genera *Firmicutes* and *Bacteroidetes* (Figure 3a). In the DF bacterial community, *Firmicutes* was the predominant phylum, with a relative abundance of up to 80.79%, followed by *Actinobacteria* (11.97%) and *Bacteroidetes* (5.51%) (Figure 3a). The predominant phylum in the DR microbiota was *Bacteroidetes* (58.82%), followed by *Firmicutes* (37.60%) and *Actinobacteria* (5.51%) (Figure 3a).

**Figure 4:**
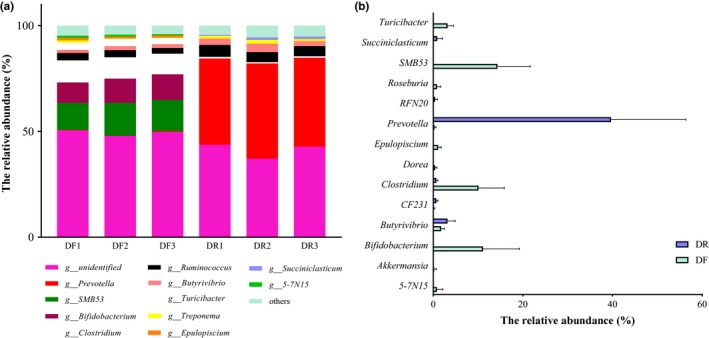
(a) Relative abundance of genus by representation at ≥ 0.001% of total sequences. (b) Relative abundance of bacterial genera between DR and DF samples (relative abundance > 1%). All data are the means of 15 samples. *q < 0.05, **q < 0.01, and ***q < 0.001

At the genus level, a total of 78 bacterial genera were identified. *Prevotella*, *Ruminococcus*, and *Butyrivibrio* were the three most abundant genera in DR of all identified genus (Figure 4a). *Bifidobacterium*, *Clostridium*, *Turicibacter*, and *Ruminococcus* were predominant in DF, accounting for 91.21% of reads (Figure 4a).

### 3.3 Differences in community composition between the DF and DR samples

The relative abundance of *Firmicutes* and *Actinobacteria* was significantly increased in the DF (*q < *0.05) (Figure 3b and Table A2), while the DR microbiota contained a higher relative abundance of *Bacteroidetes* (*q < *0.001). At the phylum level, no differences were observed in either the DR or DF communities over the three consecutive sampling days except for in the relative abundance of *Bacteroidetes*, *Firmicutes,* and *Actinobacteria* (*q < *0.001). At the phylum level, no differences were observed over three consecutive sampling days in DR bacterial communities and in DF bacterial communities (*q > *0.05).

At the genus level, the relative abundance of *Bifidobacterium*, *5‐7N15*, *Clostridium*, *Epulopiscium*, *SMB53*, *Turicibacter*, *Dorea*, *Roseburia,* and *Akkermansia* was significantly higher (*q < *0.01) in the DF community compared with the DR community, while the relative abundance of *Prevotella*, *CF231*, *Butyrivibrio*, *RFN20,* and *Succiniclasticum* was significantly lower (*q < *0.05) in DF community than DR community (Figure 4b and Table A3). There were no changes in the relative abundance of genera among different sampling days for the DR and DF microbial communities.

### 3.4 Divergence of predicted microbial functions in the DR and DF Groups

**Figure 5:**
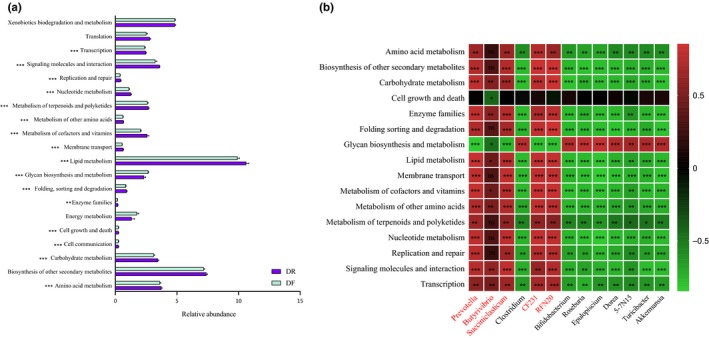
(a) Comparisons of the predicted KEGG pathways of the ruminal and fecal bacterial microbiota of dairy cows. (b) The Spearman correlation between the genus level relative abundance more than 1% and the selected predicted KEGG pathways. *0.01 < q < 0.05, **0.01 < q < 0.001, ***0.001 < q < 0.0001, ****0.0001 < q < 0.00001. The reads color genus name means the higher relative abundance in DR than those of DF

We then performed a PICRUSt analysis and found 22 KEGG pathways (level 2), 18 of which were significantly different between the DR and DF samples. Lipid metabolism (10.63%), biosynthesis of other secondary metabolites (7.40%), xenobiotics biodegradation and metabolism (4.84%), amino acid metabolism (3.74%), and signaling molecules and interaction (3.59%) were identified as the top 5 predicted functions for the DR microbiota, which were also the top predicted function for DF microbiota. Cell communication, cell growth and death, and glycan biosynthesis and metabolism were significantly higher in DF compared to the DR group (*q* < 0.05), whereas lipid metabolism, amino acid metabolism, carbohydrate metabolism, enzyme families, metabolism of cofactors and vitamins, and metabolism of other amino acids were significantly higher in DR compared to the DR group (*q* < 0.05, Figure 5a).

### 3.5 Relationship between bacterial community and predicted function

To examine the relationship between DR and DF communities, Spearman's rank correlation was used to identify linkages between predicted function and taxonomy (Figure 5b). We found that the relative abundance of *Prevotella*, *Succiniclasticum*, *CF231,* and *RFN20* spp. was positively correlated with amino acid metabolism, biosynthesis of other secondary metabolites, carbohydrate metabolism, digestive system, enzyme families, folding sorting and degradation, lipid metabolism, membrane transport, metabolism of cofactors and vitamins, metabolism of other amino acids, metabolism of terpenoids and polyketides, nucleotide metabolism, replication and repair, signaling molecules, and interaction and transcription. We also found that the relative abundance of *5‐7N15*, *Akkermansia*, *Bifidobacterium*, *Clostridium*, *Dorea*, *Epulopiscum*, *Roseburia,* and *Turicibacter* was negatively correlated with those predicted function (*q* < 0.05, Figure 5b). *Butyrivibrio* was positively correlated with carbohydrate, lipid, cofactors and vitamins, other amino acids, enzyme families, signaling molecules, and interaction and transcription*.* Cell growth and death and glycan biosynthesis and metabolism were negatively correlated with *Butyrivibrio*. Glycan biosynthesis and metabolism were positively correlated with the genera abundance in the DF community (Figure 5b).

## 4 DISCUSSION

The gastrointestinal tract of ruminant animals harbors a large number of symbiotic microbes that allow the host to acquire nutrients from its feed (Henderson et al., 2015; Roesch et al., 2007) and prevent the colonization of pathogens (Lettat et al., 2012). Ruminal fluid and fecal samples are frequently used to assess the rumen (Henderson et al., 2013; Jami, Israel, Kotser, & Mizrahi, 2013) and gut (Behr et al., 2018; Sun et al., 2018; Yang et al., 2017) microbiota of animals, respectively. In animals, intramicrobiota variation is important for health assessment, while intermicrobiota variation can be used to identify differences among the microbiota populations in the different gastrointestinal tract sections between individuals (de Oliveira et al., 2013). Here, we examined differences in the microbiota of different sites in the gut (rumen fluid and feces) and on different sampling days (three consecutive sampling days) from five lactating dairy cows using high‐throughput sequencing technology.

The bacterial microbiota in the dairy cow’ rumen were far more diverse and abundant than that of feces as reported previously (Holman & Gzyl, 2019) (Figure 1 and Table 1). In line with previous study (Mao et al., 2015; de Oliveira et al., 2013), the relative abundance of *Bacteroidetes* was higher in DR (*p* < .001, Table A2) in our study, while DF contains a greater abundance of *Firmicutes* (*p* < .001, Table A2). Similar to the study of Li et al. (2016), a greater abundance of *Clostridium*, *Turicibacter,* and *Akkermansia* and lower abundance of *Prevotella* were enriched in feces in this study.

Some studies have suggested that the large intestine provides an active site of fermentation similar to the reticulum–rumen. Some bacteria in the fecal microbiota are associated with cellulose and hemicellulose degradation, including *Ruminococcus*, *5‐7N15* (*Bacteroidaceae* family) (Khan, Saddler, Patel, Colvin, & Martin, 1980), *SMB53* (*Clostridiaceae* family, Wust, Horn, & Drake, 2011), and *Clostridium* (Ozutsumi, Hayasm, Sakamoto, Itabashi, & Benn, 2005). As a butyrate producer (Pryde, Duncan, Hold, Stewart, & Flint, 2002), Zhang et al. (2018) found that *Roseburia* was positively associated with fecal butyrate content. *Epulopiscium* bacteria are abundant in the guts of herbivorous surgeonfishes (Miyake, Ngugi, & Stingl, 2015, 2016) and ants (Russell et al., 2009). In the present study, the high relative abundance of *Epulopiscium* in the fecal microbiota might be attributed to the plant‐based diet of dairy cows.

Previous studies have reported that intestinal bacteria significantly affect the growth and health of cattle (Cui et al., 2015; Ellis et al., 2013). The genus *Bifidobacterium* (phylum *Firmicutes*) represents the primary health‐promoting functions of piglets, with preventative and protective effects against diarrhea and intestinal infections (Hu et al., 2015). Similarly, *Akkermansia* spp. have attracted growing interest in its various functions related to mucosa health, energy metabolism, and inflammation markers (Guo et al., 2017; Schneeberger et al., 2015). *Akkermansia* and *Bifidobacterium* were found to be dominant genera in the present study, which is in agreement with other studies (Li, Meng, Zhou, & Zhou, 2017; Song, Malmuthuge, Steele, & Guan, 2018; Zhang et al., 2018), and might be beneficial to bovine gastrointestinal tract health. The genus *Turicibacter* has been reported to be more highly abundant in the feces of cattle (Callaway et al., 2010; Mao, Huo, & Zhu, 2013) and is thought to be associated with dermatitis in cattle and contagious dermatitis in sheep (Evans, Brown, Hartley, Smith, & Carter, 2012; Sadet, Martin, Meunier, & Morgavi, 2007).

Thirteen genera (relative abundance of each sample > 1%) were differentially abundant between the DR and DF (Table A3). A significantly higher relative abundance of *Prevotella* was observed in the rumen fluid of dairy cows. This is in line with the known function of members of this genus, as they possess enzymes that can degrade hemicelluloses as well as starch to the short‐chain fatty acids (SCFAs) such as acetate, propionate, butyrate, and succinate (Flint & Bayer, 2008; Dodd, Mackie, & Cann, 2011). Butyrate and propionate are the most important SCFAs in dairy cattle. Members of the genus *Butyrivibrio* are butyrate producers (Mohammed et al., 2014), and *Succiniclasticum* spp. can produce propionate from succinate (van Gylswyk, 1995). This may partly explain the significantly higher abundance of *Butyrivibrio* and *Succiniclasticum* in the DR community (Figure 4b and Table A3). Two unclassified bacteria were also identified in the present and the other studies (Jewell, McCormick, Odt, Weimer, & Suen, 2015; Zhu et al., 2018), including *CF231* and *RFN20*, which were significantly abundant in the DR community.

In addition, in the present study, the predicted function of the lipid, carbohydrate, and amino acid metabolism by PICRUSt were positive and significantly correlated with *Prevotella*, *CF231*, *RFN20,* and *Succiniclasticum* (Figure 5a,b). *Butyrivibrio* has strongly correlated with predicted lipid, carbohydrate, cofactors and vitamins, and other amino acid and metabolism (Figure 5b). The SCFAs producer bacteria and metabolism function were both enriched in the DR community suggested rumen bacteria was related to the diet and rumen function of plant digestion and VFAs production.

Another important finding of this study was the lack of significant variation among samples collected over three consecutive days from each of the gut communities, indicating that dairy cows have two relatively independent and stable microbial communities in the gut when clinically healthy. This is consistent with the results of Skarlupka et al. (2019); they compared the rumen liquid and solid community from three consecutive days and found that the rumen community is limited day‐to‐day variability in the rumen microbiota. But in our study, we found that feces bacterial community were also like stable on a day‐to‐day basis, indicating that the gut microbial community is likely stable on a day‐to‐day basis.

## 5 CONCLUSIONS

Our results demonstrate striking differences in the composition of bacterial communities from the DR and DF of lactating dairy cattle, indicating that two relatively independent and stable microbial communities were existing in the gut of dairy cows. Furthermore, no significant differences in the microbiota among samples collected over three consecutive days from either the DR or DF indicated that the gut microbial community is likely stable on a day‐to‐day basis.

## CONFLICT OF INTERESTS

None declared.

## AUTHOR CONTRIBUTIONS

Shuai Huang: Conceptualization‐Equal, Formal analysis‐Lead, Software‐Lead, Visualization‐Lead, Writing‐original draft‐Lead, Writing‐review & editing‐Lead. Shoukun Ji: Conceptualization‐Equal, Formal analysis‐Supporting, Writing‐review & editing‐Supporting. Hui Yan: Software‐Supporting, Writing‐review & editing‐Supporting. Yangyi Hao: Writing‐review & editing‐Supporting. Jun Zhang: Writing‐review & editing‐Supporting. Yajing Wang: Writing‐review & editing‐Equal. Zhijun Cao: Writing‐review & editing‐Supporting. Lisheng Li: Funding acquisition‐Lead.

## ETHICS STATEMENT

The experimental protocol was reviewed and approved by the Ethical Committee of the College of Animal Science and Technology of China Agricultural University.

## Data Availability

All 16S rRNA sequences generated in this study were submitted to the NCBI Sequence Read Archive and are accessible under BioProject number PRJNA540088: https://www.ncbi.nlm.nih.gov/bioproject/PRJNA540088

## Appendix 1

**Table A1:** Ingredients and chemical composition of the diets (as dry matter basis)

Items	Content
Ingredients, kg	
Domestic alfalfa hay	15.99
Imported alfalfa hay	4.13
Corn silage	21.15
Ground corn	1.95
Corn‐steam flaked	20.73
Extruded soybean meal	2.15
Soybean meal	11.62
Soybean hulls	11.66
Whole cottonseed	4.00
Premix	6.62
Chemical composition, %	
DM as feed	56.4
Crude protein	15.42
Fat	4.85
NDF	31.02
ADF	20.11
Ash	7.63
Ca	0.92
P	0.41

Abbreviations: ADF, acid detergent fiber; Ca, calcium; NDF, neutral detergent fiber; P phosphorus.

**Table A2:** The relative abundance of major bacterial phyla (>1%) between rumen fluid and feces

Phylum		Day 1	Day 2	Day 3	*q*‐value for day	*q*‐value (DF versus DR)
*Bacteroidetes*	DR	58.07	57.74	60.05	ns	***
	DF	6.72	4.02	5.79	ns	
	*q*‐value	***	***	***		
*Firmicutes*	DR	38.69	37.87	36.24	ns	***
	DF	79.68	82.8	79.91	ns	
	*q*‐value	***	***	***		
*Spirochaetes*	DR	1.22	1.67	1.02	ns	**
	DF	1.09	0.22	0.15	ns	
	*q*‐value	ns	*	ns		
*Actinobacteria*	DR	0.33	0.53	0.28	ns	**
	DF	10.39	11.97	12.70	ns	
	*q*‐value	*	***	***		
*Proteobacteria*	DR	0.27	0.43	0.28	ns	*
	DF	0.26	0.17	0.11	ns	
	*q*‐value	ns	ns	ns		

Note

Day 1: sample from the first sampling day; day 2: sample from the second sampling day; day 3: sample from the third sampling day.

Abbreviations: DF, feces samples; DR, rumen fluid samples.

**q* value < 0.05, ***q* value < 0.01, ****q* value < 0.001, ns means *q* > 0.05.

**Table A3:** Dominant genera (>1%) calculated from collected samples of ruminal fluid and feces

Phyla	Genera		Day 1	Day 2	Day 3	*q*‐value for day	*q*‐value (DF versus DR)
*Actinobacteria*	*Bifidobacterium*	DF	9.73	11.40	12.14	ns	***
		DR	0.02	0.03	0.02	ns	
		*q*‐value	ns	*	*		
*Bacteroidetes*	*Prevotella*	DF	0.30	0.17	0.38	ns	***
		DR	32.34	40.49	46.15	ns	
		*q*‐value	ns	***	***		
*Bacteroidetes*	*5‐7N15*	DF	1.00	0.90	0.54	ns	**
		DR	0	0	0.001	ns	
		*q*‐value	ns	*	ns		
*Bacteroidetes*	*CF231*	DF	0.29	0.08	0.14	ns	**
		DR	0.55	0.71	0.87	ns	
		*q*‐value	ns	***	***		
*Firmicutes*	*Butyrivibrio*	DF	1.51	1.93	1.82	ns	*
		DR	2.95	4.26	2.33	ns	
		*q*‐value	ns	ns	ns		
*Firmicutes*	*Clostridium*	DF	10.46	10.10	9.76	ns	***
		DR	0.72	0.66	0.68	ns	
		*q*‐value	ns	***	***		
*Firmicutes*	*Epulopiscium*	DF	1.26	0.93	1.11	ns	***
		DR	0	0.001	0.001	ns	
		*q*‐value	ns	***	***		
*Firmicutes*	*Ruminococcus*	DF	3.47	3.33	2.67	ns	ns
		DR	4.43	6.31	4.20	ns	
		*q*‐value	ns	ns	ns		
*Firmicutes*	*SMB53*	DF	12.64	15.63	14.72	ns	***
		DR	0.01	0.01	0.01	ns	
		*q*‐value	ns	***	***		
*Firmicutes*	*Turicibacter*	DF	3.33	3.37	2.92	ns	***
		DR	0.002	0.005	0.003	ns	
		*q*‐value	ns	***	*		
*Firmicutes*	*Dorea*	DF	0.49	0.52	0.27	ns	***
		DR	0	0.001	0	ns	
		*q*‐value	ns	***	*		
*Firmicutes*	*Mogibacterium*	DF	0.52	0.60	0.61	ns	ns
		DR	0.25	0.47	0.22	ns	
		*q*‐value	ns	ns	ns		
*Firmicutes*	*Oscillospira*	DF	0.85	0.83	0.87	ns	ns
		DR	0.76	0.55	0.59	ns	
		*q*‐value	ns	ns	ns		
*Firmicutes*	*RFN20*	DF	0.001	0	0	ns	**
		DR	0.34	0.38	0.56	ns	
		*q*‐value	ns	*	*		
*Firmicutes*	*Roseburia*	DF	0.96	0.94	0.65	ns	**
		DR	0.01	0.01	0.01	ns	
		*q*‐value	ns	***	***		
*Firmicutes*	*Succiniclasticum*	DF	0.001	0.002	0.003	ns	***
		DR	0.40	1.19	1.11	ns	
		*q*‐value	ns	ns	*		
*Spirochaetes*	*Treponema*	DF	1.09	0.22	0.15	ns	ns
		DR	0.98	1.28	1.44	ns	
		*q*‐value	ns	ns	*		
*Verrucomicrobia*	*Akkermansia*	DF	0.06	0.05	0.48	ns	***
		DR	0	0	0	NA	
		*q*‐value	ns	*	*		

Note

Day 1: sample from the first sampling day; day 2: sample from the second sampling day; day 3: sample from the third sampling day.

Abbreviations: DF, feces samples; DR, rumen fluid samples.

**q* value < 0.05, ***q* value < 0.01, ****q* value < 0.001, ns means *q* > 0.05.
